# Epidemiology of multimorbidity within the Brazilian adult general population: Evidence from the 2013 National Health Survey (PNS 2013)

**DOI:** 10.1371/journal.pone.0171813

**Published:** 2017-02-09

**Authors:** Magdalena Rzewuska, João Mazzoncini de Azevedo-Marques, Domenica Coxon, Maria Lúcia Zanetti, Ana Carolina Guidorizzi Zanetti, Laercio Joel Franco, Jair Lício Ferreira Santos

**Affiliations:** 1 Community Health Postgraduate Program, Ribeirão Preto School of Medicine, University of São Paulo, Ribeirão Preto, São Paulo, Brazil; 2 Department of Social Medicine, Ribeirão Preto School of Medicine, University of São Paulo, Ribeirão Preto, São Paulo, Brazil; 3 Centre for Population Health Sciences, University of Edinburgh, Lothian, Scotland; 4 WHO Collaborating Centre for Nursing Research Development, Ribeirão Preto College of Nursing, University of São Paulo, Ribeirão Preto, São Paulo, Brazil; Yokohama City University, JAPAN

## Abstract

Middle-income countries are facing a growing challenge of adequate health care provision for people with multimorbidity. The objectives of this study were to explore the distribution of multimorbidity and to identify patterns of multimorbidity in the Brazilian general adult population. Data from 60202 adults, aged ≥18 years that completed the individual questionnaire of the National Health Survey 2013 (Portuguese: “Pesquisa Nacional de Saúde”–“PNS”) was used. We defined multimorbidity as the presence of two or more chronic conditions, including self-reported diagnoses and responses to the 9-item Patient Health Questionnaire for depression. Multivariate Poisson regression analyses were used to explore relationship between multimorbidity and demographic factors. Exploratory tetrachoric factor analysis was performed to identify multimorbidity patterns. 24.2% (95% CI 23.5–24.9) of the study population were multimorbid, with prevalence rate ratios being significantly higher in women, older people and those with lowest educational level. Multimorbidity occurred earlier in women than in men, with half of the women and men aged 55–59 years and 65–69 years, respectively, were multimorbid. The absolute number of people with multimorbidity was approximately 2.5-fold higher in people younger than 65 years than older counterparts (9920 *vs* 3945). Prevalence rate ratios of any mental health disorder significantly increased with the number of physical conditions. 46.7% of the persons were assigned to at least one of three identified patterns of multimorbidity, including: “cardio-metabolic”, “musculoskeletal-mental” and “respiratory” disorders. Multimorbidity in Brazil is as common as in more affluent countries. Women in Brazil develop diseases at younger ages than men. Our findings can inform a national action plan to prevent multimorbidity, reduce its burden and align health-care services more closely with patients’ needs.

## Introduction

Management of the increasingly common problem of multimorbid chronic diseases is a major global healthcare challenge [1]. Multimorbidity is assisted with functional decline, an increased risk of mortality and an increased use of healthcare resources [2–5]. Compared to multiple chronic physical diseases, physical and mental health comorbidity is associated with even greater functional decline [6]. Healthcare approaches focused on single disease are unlikely to effectively coordinate healthcare for the complex needs of people with multimorbidity [7–10]. Whilst multimorbidity is known to increase with age and socioeconomic deprivation, estimates of the prevalence of multimorbidity are heterogeneous; most studies have counted small numbers of morbidities, are focused on older people and hospital populations, physical health problems and high-income countries [11–13].

The World Health Organization´s (WHO) global action plan for prevention and control of chronic diseases emphasises low and middle-income countries, where the majority of world population lives, socioeconomic deprivation is more common and where 80% all deaths due to chronic disease occur [14]. Recent, first multi-national investigation in low and middle-income countries reported prevalence rates of multimorbidity in older people that varied widely [13], suggesting that multimorbidity needs better understanding in the context of country-specific characteristic, such as demographic structure, disease burden and health system.

Brazil is a rapidly developing middle-income country with the world’s fifth largest population [15] and the rising incidence of chronic non-communicable diseases [16]. The estimated economic cost due to deaths from chronic non-communicable diseases and productivity losses due to absenteeism and presenteeism in Brazil in 2015 equated to a total of 5.4% (US$129.8 billions) of Gross Domestic Product [17]. To meet the demand for cost effective, efficient, safe, high quality provision of comprehensive care, Brazil has progressively strengthened the Family Health Programme and recently established operation norms for a regionalised network of health services [18, 19]. The coordination of the regionalised network of health services remains complex as it includes distinct but interconnected public and private sectors [19]. A specific action plan for prevention and control of multimorbidity through this highly complex system is currently lacking and its development is hindered partially by limited evidence. In Brazil, multimorbidity occurrence and patterns has been studied only in relatively small samples of the general population of one city [20], in older people [21] and in women [22]. Even less is known about mental health comorbidity specifically in Brazil [23], and this is despite the 12-month prevalence rate of major depressive episode in Brazil being estimated to be the highest in the world [24].

Better understanding of multimorbidity in Brazil can inform interventions to prevent and control multimorbidity, reduce its burden, and align health-care services more closely with patients’ needs in Brazil and in comparable middle-income countries. We aimed to use a large, nationally representative sample of Brazilian adults to explore the distribution of multimorbidity and to identify patterns of multimorbidity of chronic physical and mental health conditions.

## Materials and methods

### Study design and participants

This study is a cross-sectional analysis of the National Health Survey (Portuguese: “Pesquisa Nacional de Saúde”–“PNS”), Brazil 2013. The study sample and data collection have been detailed elsewhere [25, 26]. Ethical approval was gained for this study from the National Commission for Ethics in Research (CONEP) of the National Health Council (CNS), Ministry of Health (no. 328 159, 26 June 2013).

The PNS in Brazil is a community-based nationwide survey representative for macro-regions of Brazil and included federated and capital units, metropolitan areas and the rest of the federal units of the country. The survey is a part of the Integrated Household Surveys (SIPD) conducted by the Ministry of Health and Brazilian Institute of Geography and Statistics (IBGE). The primary sampling units are census tracts based on the 2010 census and randomly selected from the IBGE national master sampling plan. Within each census tract households were randomly selected from a national registry of addresses. The data was collected between August 2013 and February 2014. All the data collection process of PNS was performed with the use of handheld computers (Personal Digital Assistant—PDA), programmed for critical process variables. All PNS datasets are available from the IBGE´s website.

Cluster sampling was conducted over three stages: census sectors or the set of these sectors form the Primary Sampling Units (PSU), the households were the units of the second stage and adult residents (aged 18 years or more) formed the units of the third stage. The survey included the following three questionnaires: the household questionnaire, related to the characteristics of the household; the questionnaire related to all residents of the household; and the individual questionnaire, answered by an adult resident aged 18 or more. The selection of the PSU sub-sample was done with simple random sampling. The final sample size was adjusted based on the values of the design effect (DEFF), with a total number of 81167 households being estimated in the sampling, of those 64348 households participated in interviews, which results in 79% response rate. Further, 60202 individuals answered to the individual questionnaire.

Sample weights were defined for the PSU, the households and all of their residents. A weight for the selected resident was calculated considering the weight of the related household, the probability of selecting the resident, adjustments of non-response by sex and adjustment of population totals by sex and age, estimated with the weight of all residents combined. All PNS datasets are available from the IBGE website, located at: http://www.ibge.gov.br/home/estatistica/populacao/pns/2013/default_microdados.shtm.

### Outcomes

The individual questionnaire included a question on chronic back problem (“Do you have any chronic back problem, such as chronic back pain or neck, low back pain, sciatica, problems in the vertebrae or disc?“). Other diseases were based on questions: “Has a doctor given you the diagnosis of […]?“. Included were arthritis/rheumatism, cancer, diabetes, stroke, cardiovascular disorders including heart attack, angina, heart failure or other; high blood cholesterol, hypertension, asthma, chronic obstructive pulmonary disease including emphysema, chronic bronchitis or other; musculoskeletal disorders related to work, chronic renal insufficiency and “other chronic/long-term physical or mental condition (six or more months)”. The PNS2013 data on prevalence rates of specific chronic physical health disorders can be found published elsewhere [27].

Mental health morbidities included self-reported diagnosis of psychiatric disorders, based on questions: “Has a doctor or mental health professional (such as psychiatrist or psychologist) given you the diagnosis of mental illness, such as […]?” We have included this way ascertained schizophrenia, obsessive compulsive disorder, bipolar disorder or other mental health disorder. Depression was ascertained with the 9-item Patient Health Questionnaire (PHQ-9) using a diagnostic algorithm for major depressive episode, which was found to be reliable and valid screening tool for major depressive episodes in a sample of the general population in Brazil [28]. The PNS2013 estimates of prevalence rates of those specific mental health disorders has been published elsewhere [27, 29].

Predictor variables included: sex (male/female), age (18–24, 25–44, 45–64, 65–84 and ≥85), educational levels (no formal education/ incomplete fundamental, complete fundamental/incomplete intermediate, complete intermediate/incomplete superior and complete superior) and country region (North/Northeast/Midwest/South and Southeast). There was no missing data.

### Analysis

For descriptive purposes we used unweighted frequencies, but proportions incorporated sampling weights and the complex sampling design. Poisson regression analyses with robust variance were conducted to examine the relationship between multimorbidity (defined as the presence of two or more of morbidities in one person) and demographic variables (age, sex, country regions and educational levels). Results are reported as adjusted prevalence rate ratios with 95% CI, which are easier to interpret and communicate to non-specialists than the odds ratio [30]. In addition to the listed demographic variables, estimates were adjusted according to availability for factors that could potentially influence the likelihood of being diagnosed, such as: disability (*i*.*e*. restricted activity days in the past two weeks due to ill health) and access to health services (*i*.*e*. number of medical consultations in the past year, having a private healthcare plan and being registered with a Family Health Programme unit). Results incorporate sampling weights and the complex sampling design.

Next, we conducted identical analyses to examine the link between the number of physical conditions and any mental health condition (adjusted for age, sex, country regions and educational levels, restricted activity days in the past two weeks due to ill health, number of medical consultations in the past year, having a private healthcare plan). Being registered with a Family Health Programme unit was excluded this time from this model, given to our understanding mental health conditions are unlikely to be diagnosed in primary care in Brazil [31–35]. Multimorbidity patterns were analysed through exploratory factor analysis (EFA), as per previous studies [13, 20, 36, 37]. This technique summarizes the correlations in a series of variables with the aim of exploring the underlying structure of the data. We used the iterated EFA to fit the common factor model to the data. Here the initial estimates of the communalities would be the squared multiple correlation coefficients, but the solution would then be iterated and if convergent obtain better estimates [38]. Due to the binary nature of the variables, we used a tetrachoric correlation matrix assuming that the disease has a progressive nature until reach diagnosis [39]. The Keiser-Guttman and the scree test method is the most commonly used to decide the number of factors [20, 36, 37, 40], however due to its limitations the former is not recommended [40–42]. Therefore we used a combined approach to select number of factors, as recommended, specifically we chose two statistical methods (*i*.*e*. the scree test and the parallel analysis [13]) and a pragmatic evaluation, including minimum explained variance (≥ 10% by each factor), cumulative variance by retained factors (≥70%) and, at least, two variables in the final solution [20]. The Keiser-Meyer-Olkin (KMO) method was used to estimate adequacy of the sample, with value over 0.5 suggesting a satisfactory factor analysis [43]. Oblique rotation was performed to provide easier interpretation of the factor analysis. For consistency with previous comparable research a condition was included in the factor if its corresponding rotated factor loading was above 0.25 (indicting a strong association) [13, 37]. STATA statistical software version 11.0 (StataCorp, College Station, TX) was used for all analyses.

## Results

The study population was likely to be aged 25–44, live in the South of Brazil and had none or incomplete or fundamental educational background (Table 1). Men and women were equally represented. 49.1% (95% CI 48.3–49.9) of the sample had one or more morbidities and 24.2% (95% CI 23.5–24.9) were multimorbid. Prevalence rates of multimorbidity increased with age, but after the age of 65 years started to level off (Table 1). 55.9% of people aged 45–59 years had one or more morbidity and 53.2% people aged 65–69 years were multimorbid (data not shown). In absolute terms, multimorbidity was 2.5-fold higher in people younger than 65 years (9920 *vs* 3954). Multimorbidity prevalence was higher in women than men (Table 1). 21.1% of men and 29.3% of women aged 20–24 years already had one or more morbidity (S1 Fig). Most women aged 40–44 years and most men aged 50–54 years had one or more morbidity. Half of the women and men aged 55–59 years and 65–69 years, respectively, were multimorbid. An adjusted Poisson regression model showed that multimorbidity varied across the country regions and was significantly higher in women, older people and those with lowest educational level (Table 1).

**Table 1:** Multimorbidity (≥2 conditions) in the Brazilian adult general population (n = 60202).

	n [%]	Proportion with multimorbidity [95% CI]	Adjusted ^a^ PRR of multimorbidity [95% CI]
Gender			
Male	25920 [47.1]	19.0 [18.2–19.9]	1
Female	34282 [52.9]	28.8 [27.9–29.7]	1.36 [1.29–1.43]
Age, years			
18–24	7823 [15.9]	5.5 [4.6–6.7]	1
25–44	26740 [40.8]	13.2 [12.5–14.0]	2.23 [1.84–2.70]
45–64	17927 [31.0]	36.2 [34.9–37.2]	5.48 [4.52–6.66]
65–84	7079 [11.2]	54.7 [52.5–56.7]	7.45 [6.13–9.05]
≥85	633 [1.1]	53.7 [47.4–59.8]	6.82 [5.65–9.00]
Educational levels			
No education/incomplete fundamental	25524 [38.9]	33.6 [32.4–34.3]	1
Complete fundamental/incomplete intermediate	9815 [15.6]	19.7 [18.2–21.2]	0.92 [0.94–0.98]
Complete intermediate/incomplete superior	20632 [32.8]	15.8 [14.9–16.7]	0.78 [0.73–0.84]
Complete superior	8337 [12.7]	22.8 [20.5–24.7]	0.83 [0.76–0.91]
Country regions			
North	12536 [7.4]	17.3 [16.0–18.7]	1
Northeast	18305 [26.6]	21.5 [20.5–22.6]	1.07 [0.98–1.16]
Midwest	7519 [7.4]	22.5 [21.2–23.9]	1.11 [1.02–1.22]
South	14294 [43.8]	25.7 [24.4–27.0]	1.17 [1.08–1.28]
Southeast	7548 [14.8]	28.9 [27.2–30.7]	1.31 [1.19–1.43]
Restricted activity days (past 2 weeks)			1.04 [1.03–1.04]
Number of medical consultations (past year)			1.04 [1.03–1.04]
Registered with a family health care unit			1.07 [1.01–1.12]
Having a private healthcare plan			1.18 [1.12–1.25]

Unweighted frequencies, proportions with 95% CI (confidence interval) and Prevalence Rate Ratios (PRR) with 95% CI incorporating appropriate weights to control for the complex sample design.

^a^ Estimates were adjusted for other listed variables in the model.

Overall, 4.8% (95% CI 4.5, 5.1%) of the study population had any mental health disorder. The proportion of people with any mental health disorders increased with number of physical conditions, with up to 23.4% in people with five or more physical health problems (Table 2). However, as much as 74.6% (95% CI 71.8, 77.1%) of people with any mental health disorder had one or more chronic physical health problem. Prevalence of any mental health disorder was highest in women aged 24–44 in general and in men 45–64 and ≥85 years with four or more physical health conditions (S2 Fig). Likewise for multimorbidity prevalence rate ratios of any mental health disorder varied significantly across the country regions and were higher in women and people with lowest educational level (Table 2), but overall age were not significantly associated with having any mental health disorder.

**Table 2:** Any mental health disorder by sex, age, educational level, country region and number of physical disorders (n = 60202).

	Percentage with any mental health disorder [95% CI]	Adjusted ^a^ PPR of any mental health disorder [95% CI]
Gender		
Male	3.1 [2.8–3.5]	1
Female	6.3 [5.9–6.8]	1.63 [1.43–1.87]
Age squared		0.99 [0.99–1.00]
Educational levels		
No education/incomplete fundamental	6.3 [6.8–6.9]	1
Complete fundamental/incomplete intermediate	5.4 [4.6–6.2]	0.86 [0.72–1.04]
Complete intermediate/incomplete superior	3.3 [3.0–3.8]	0.65 [0.56–0.76]
Complete superior	4.1 [3.2–5.2]	0.77 [0.60–0.98]
Country regions		
North	3.2 [2.8–3.8]	1
Northeast	4.5 [4.0–5.0]	1.24 [1.04–1.49]
Midwest	4.7 [4.1–5.4]	1.33 [1.08–1.62]
South	5.1 [4.5–5.7]	1.39 [1.14–1.70]
Southeast	5.6 [4.9–6.5]	1.48 [1.22–1.78]
Number of physical disorders		
0	2.4 [2.1–2.6]	1
1	5.1 [4.5–5.8]	2.06 [1.71–2.47]
2	6.5 [5.6–7.4]	2.47 [2.02–3.02]
3	11.6 [9.9–13.5]	4.09 [3.25–5.16]
4	15.5 [12.7–18.9]	5.01 [3.85–6.53]
≥5	23.4 [19.4–28.0]	6.59 [4.91–8.86]
Restricted activity days (past 2 weeks)		1.09 [1.08–1.10]
Number of medical consultations (past year)		1.03 [1.02–1.04]
Having a private healthcare plan		0.84 [0.72–0.98]

Proportions with 95% CI (confidence interval) and Prevalence Rate Ratios (PRR) with 95% CI incorporate appropriate weights to control for the complex sample design.

^a^ Estimates were adjusted for other listed variables in the model.

**Fig 1:**
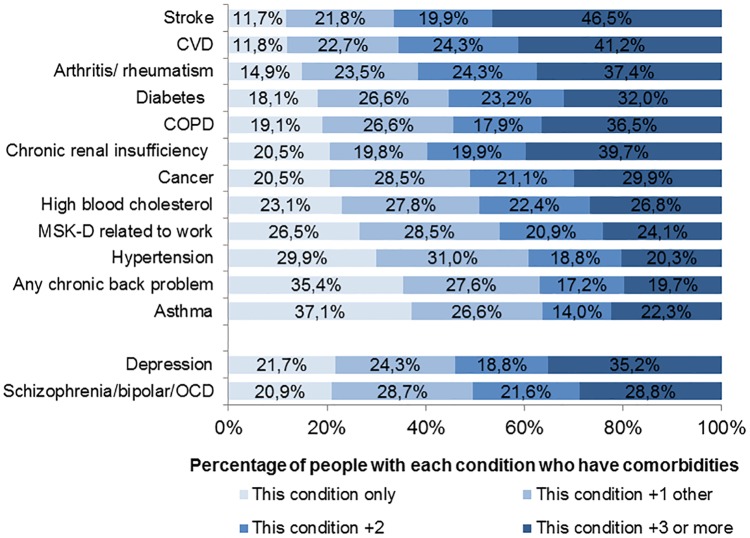
Numbers of conditions comorbid with each specific chronic disorder. Abbreviations: COPD, chronic obstructive pulmonary disorder; CVD, cardiovascular disorders; OCD, obsessive-compulsive disorder; MSK-D, musculoskeletal disorder. Proportions incorporate appropriate weights to control for the complex sample design.

Fig 1 shows numbers of conditions comorbid with each specific chronic disorder. 88.3% of people with stroke had one comorbidity and as many as 66.5% had two or more comorbidities. For comparison, 62.9% of people with asthma had one comorbid disorder and 26.3% had two or more comorbidities. People with major depressive disorder had a comparable prevalence rate of comorbidity to people with severe mental health disorders such as schizophrenia, obsessive-compulsive disorder or bipolar disorder. The most prevalent comorbid condition was hypertension, any chronic back problem, high blood cholesterol and arthritis or rheumatism (S3 Fig).

The exploratory tetrachoric factor analysis revealed three relevant patterns. The KMO coefficient was 0.83 that corresponds to a satisfactory sampling adequacy of the data. The screen plot with the parallel analysis can be found in supplementary appendix (S4 Fig). Cumulative variance after three factors retention was 99% (Factor 1: 67%, Factor 2: 21%, Factor 3: 11%). Factor loadings after oblique rotation can be found in Table 3. The first factor can be interpreted as “cardio-metabolic” (Men: 26.7% and Women: 34.5%). The second factor was named “musculoskeletal-mental” (Men: 27.0% and Women: 38.1%) and the third “respiratory” (Men: 4.8% and Women: 6.3%). 46.7% of the persons were assigned to at least one of the three multimorbidity patterns.

**Table 3:** Factor loadings after oblique rotation [>0.25] (n = 60202).

	Factor 1	Factor 2	Factor 3
Arthritis or rheumatism		0.56	
Cancer			
Diabetes	0.67		
Stroke	0.60		
CVD	0.50		
High blood cholesterol	0.48	0.27	
Hypertension	0.87		
Asthma			0.66
COPD			0.89
MSK-D related to work		0.60	
Any chronic back problem		0.69	
Chronic renal insufficiency		0.30	
Schizophrenia/Bipolar/OCD		0.27	
Depression		0.38	

Abbreviations: CVD, cardiovascular disorders; COPD, chronic obstructive pulmonary disease; OCD, obsessive-compulsive disorder

## Discussion

Our analysis of a large, nationally representative dataset shows that overall as many as 24.2% of the community-based Brazilian adults have multimorbidity of chronic conditions, but estimates vary across age, sex, educational levels and country regions. Our overall estimate is comparable to that reported for 1751841 people registered with 314 medical practise in Scotland (23.2%) [2]–to date the largest sample used for multimorbidity analysis and perhaps one of the most cited work on epidemiology of multimorbidity—suggesting that multimorbidity in the general adult Brazilian population reached the figures of richer countries. Our estimate for the Southern region of Brazil [25.7% (95% CI 24.4, 27.0)] is comparable to the estimate previously reported for that population in a southern Brazilian city (29.1%) [20]. Likewise in Finland, India, Poland, Russia, South Africa and Spain, prevalence of multimorbidity was highest in people with lower education [13]. Previous research showed prevalence of multimorbidity is highest in older people and increases with age [2, 4, 13, 20], this pattern we found in the Brazilian population.

What is new or less described in the literature is that in Brazil people develop morbidities and multimorbidity at younger age than people in more affluent countries [2, 4] and women 10 years faster than men. Interestingly, in the Scottish population multimorbidity prevalence continued substantially growing after age of 65 years [2], in several low and middle income countries [13] and in our population multimorbidity prevalence rates levelled off after age of 65–70 years. As per previous research, women had consistently higher prevalence rates of mental health problems [2], but in our research it was not associated with age. A detailed exploration revealed possible critical periods, with highest prevalence rates of any mental health disorder at aged 24–44 years in women and 45–64 and ≥85 years in men. Overall, prevalence of any mental health disorder increased with number of physical health problems, as shown in the previous research [2]. We further found that while 1 in 4 people with one or more physical health problems had mental health comorbidity, 3 in 4 people with mental health problem had comorbid physical health problem. Whilst our findings confirm that overall hypertension is the most prevalent comorbidity in the middle-income countries [13, 20], we further found that it is people with stroke that have the highest prevalence of comorbidity and people with asthma have the lowest. The identified three patterns of multimorbidity (“cardio-metabolic”, “musculoskeletal-mental” and “respiratory”), resemble those found by Garin and colleagues [13] across middle and low-income countries (named: “metabolic”, “mental-articular” and “respiratory”) using a similar methodology.

We analysed a large sample of general adult population representative to macro-regions of Brazil, the world’s fifth largest population, including a range of chronic diseases and demographic factors. The PNS survey was very well designed and had a good response rate [25, 26]. One study limitation is unobtainability of data on socioeconomic classes. The PNS questionnaire included questions on income, but this data was not made available to the public domain. Socioeconomic classes were previously found to be significantly associated with multimorbidity in several countries including Brazil [2, 13, 20], albeit the evidence is not consistent across all countries [13].

A major methodological consideration is the definition and scope of chronic condition used. A systematic review of multimorbidity studies found that the most common source of data were self-reports and the most common cut off point for multimorbidity was two conditions [44], likewise in our study. Chronic morbidity estimates based upon self-reports may lead to underestimation of multimorbidity, with one study showing that more than half of the respondents with chronic diseases failed to report at least one disease [45]. Another study suggests that patients' self-reports on selected chronic diseases are fairly accurate, with the exceptions of atherosclerosis and arthritis, where men tend to over-report stroke and underreport malignancies and arthritis and women tend to over-report malignancies and arthritis [46]. Overall, either over-reporting or under-reporting may have occurred [45, 46]. Nevertheless, we considered the PNS data most useful for the study purpose, because reliable estimates of diagnosis are unobtainable on a national level through medical records in Brazil, due to medical records remaining often non-computerized and non-integrated, common self-referrals to secondary and tertiary care services, and simultaneous access to both public and private health systems. To date no nationally representative survey in Brazil used symptom-based algorithms or measurements to assess chronic conditions.

Another issue is a possible impact of gender on likelihood of being diagnosed with a disease. The consultation behaviour of the Brazilian population is under-researched, but one large study suggests that women in Brazil may be more likely to consult then men [47]. Existing evidence on gender differences in medical consultation from other countries is mixed [48–50], and evidence comparing consultation patterns in men and women with similar morbidity is inconsistent [51, 52]. Overall, we cannot eradicate the possibility that the reported gender difference in multimorbidity was linked to gender differences in consultation patterns. Nevertheless, we attempted to minimise this issue by adjusting Poisson models for factors that could affect consultation behaviour.

Primary care plays a vital role in the prevention and control of chronic disease worldwide [53–55]. The need for strengthening multi-disease approaches to healthcare, including primary care services is well recognized [2, 9]. In more affluent countries management of multimorbidity remains challenging due to: disorganisation and fragmentation of healthcare [56]; the inadequacy of guidelines and evidence-based medicine [56]; challenges in delivering patient-centred care [56]; barriers to shared decision-making [56] and limited evidence on interventions designed to improve outcomes in people with multimorbidity [57]. In Brazil, those problems are likely to hold true due to complexity and numerous limitations of the healthcare system. Currently, the management of multimorbidity in primary care in Brazil is not an established priority. The National Program for Access and Quality Improvement in Primary Care (PMAQ) was created in 2011 [58]. Diabetes, hypertension and drug/alcohol are the chronic conditions included in the PMAQ´s quality of outcome framework [58]. Our study emphasises a need to consider expanding the focus of primary health care teams beyond those conditions to align health-care services more closely with multimorbid patients’ needs. To meet this demand effectively a substantial increase in initiatives to address fundamental research questions is needed, such as the impact of chronic non-communicable diseases on healthcare spending [59] and integration of healthcare [60], as it is happening increasingly in more affluent countries.

## Conclusions

Our results challenge the existing approach focused on patients with only one disease, currently dominating clinical guidelines and the health care system in Brazil. In order to meet the healthcare needs of the diverse Brazilian population with prevalent multimorbidity of chronic conditions, a national action plan will be needed to support primary care teams in provision of person-centred, continues and collaborative care for people with multimorbidity. Our findings suggest that beneficiaries of this approach are most likely to be women for whom multimorbidity happens earlier, is more common and more frequently is associated with mental health disorder. Whilst multimorbidity is more common in older people, we showed that strategies to prevent and control chronic conditions in Brazil should not be limited to older adults.

## Supporting information

S1 Fig Number of chronic disorders by age-group in women and men. (TIF)

S2 Fig Prevalence of mental illness by age-group and number of physical disorders in women and men. (TIF)

S3 Fig Selected comorbidities in all named disorders included in the study. Abbreviations: COPD, chronic obstructive pulmonary disorder; CVD, cardiovascular disorders; OCD, obsessive-compulsive disorder; MSK-D, musculoskeletal disorder. To interpret the graph chose a condition in the left column and for the total number of patients with this condition check the percentage of those who have the specified comorbid condition listed below. Proportions incorporate appropriate weights to control for the complex sample design. Background colours darken with increasing proportion.
(TIF)

S4 Fig The results of the screen test and the parallel analysis. The dashed line (parallel analysis) indicates a possibility that there are eleven factors, which is an unreasonably high number of factors. The light grey line shows the points that approximate a straight line (scree test). The scree plot suggests either three or five factors due to the way the slope levels off twice. Highlighted points on the straight line are selected factors.
(TIF)
