# Supplementary figures and images for: Epidemiology of multimorbidity within the Brazilian adult general population: Evidence from the 2013 National Health Survey (PNS 2013)
Source: PLoS One. 2017 Feb 9;12(2):e0171813. doi: 10.1371/journal.pone.0171813 (PMC5300133; doi:10.1371/journal.pone.0171813)

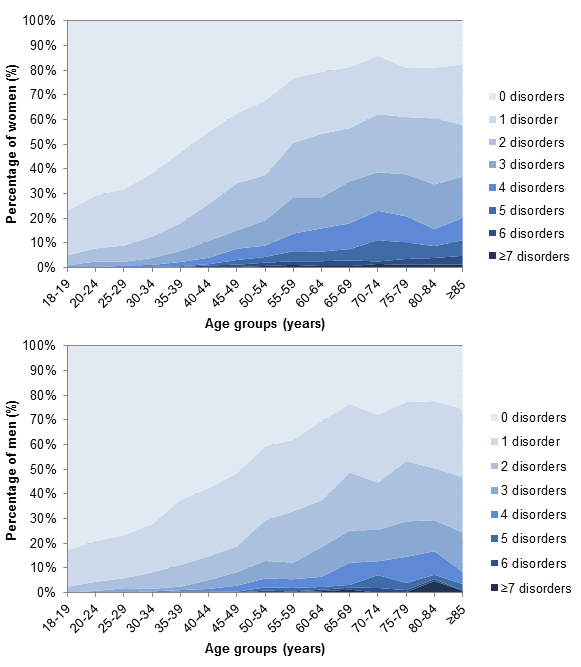

Supplement: S1 Fig — (TIF) [file pone.0171813.s001.tif]

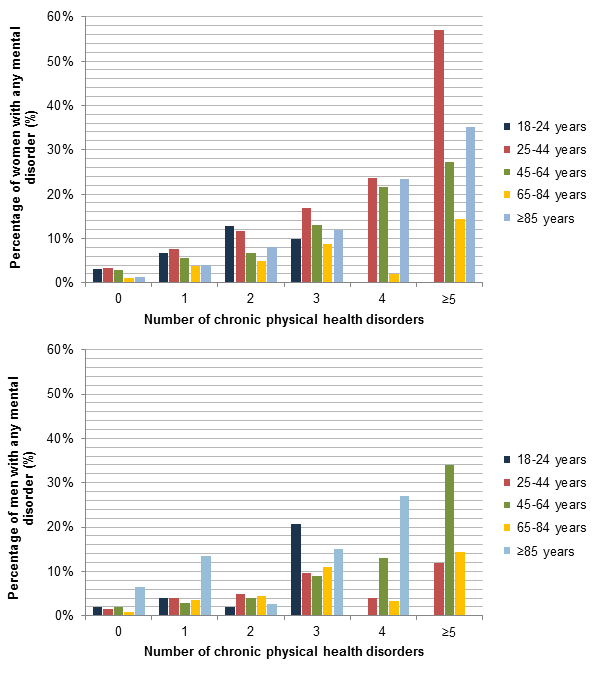

Supplement: S2 Fig — (TIF) [file pone.0171813.s002.tif]

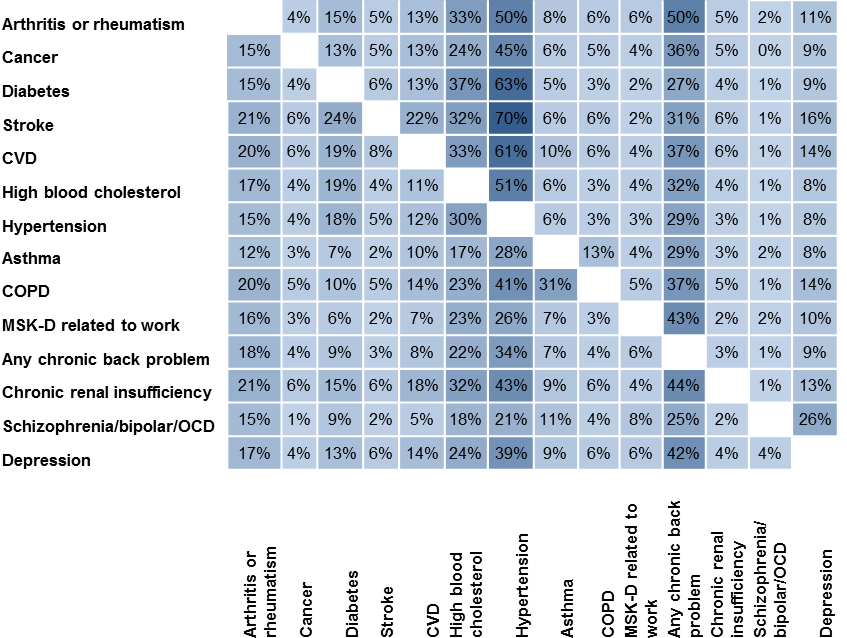

Supplement: S3 Fig — Abbreviations: COPD, chronic obstructive pulmonary disorder; CVD, cardiovascular disorders; OCD, obsessive-compulsive disorder; MSK-D, musculoskeletal disorder. To interpret the graph chose a condition in the left column and for the total number of patients with this condition check the percentage of those who have the specified comorbid condition listed below. Proportions incorporate appropriate weights to control for the complex sample design. Background colours darken with increasing proportion. (TIF) [file pone.0171813.s003.tif]

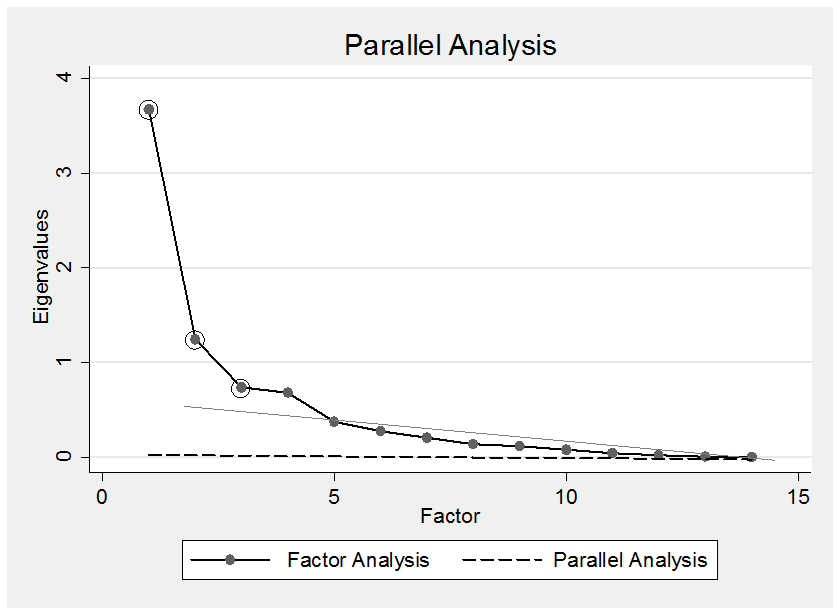

Supplement: S4 Fig — The dashed line (parallel analysis) indicates a possibility that there are eleven factors, which is an unreasonably high number of factors. The light grey line shows the points that approximate a straight line (scree test). The scree plot suggests either three or five factors due to the way the slope levels off twice. Highlighted points on the straight line are selected factors. (TIF) [file pone.0171813.s004.tif]
